# Sensitive Determination of Onco-metabolites of D- and L-2-hydroxyglutarate Enantiomers by Chiral Derivatization Combined with Liquid Chromatography/Mass Spectrometry Analysis

**DOI:** 10.1038/srep15217

**Published:** 2015-10-13

**Authors:** Qing-Yun Cheng, Jun Xiong, Wei Huang, Qin Ma, Weimin Ci, Yu-Qi Feng, Bi-Feng Yuan

**Affiliations:** 1 Key Laboratory of Analytical Chemistry for Biology and Medicine (Ministry of Education), Department of Chemistry, Wuhan University, Wuhan 430072, China; 2 Key Laboratory of Genomic and Precision Medicine, Beijing Institute of Genomics, Chinese Academy of Sciences, Beijing, 100101, China

## Abstract

2-hydroxyglutarate (2HG) is a potent competitor of α-ketoglutarate (α-KG) and can inhibit multiple α-KG dependent dioxygenases that function on the epigenetic modifications. The accumulation of 2HG contributes to elevated risk of malignant tumors. 2HG carries an asymmetric carbon atom in its carbon backbone and differentiation between D-2-hydroxyglutarate (D-2HG) and L-2-hydroxyglutarate (L-2HG) is crucially important for accurate diagnosis of 2HG related diseases. Here we developed a strategy by chiral derivatization combined with liquid chromatography-electrospray ionization-tandem mass spectrometry (LC-ESI-MS/MS) analysis for highly sensitive determination of D-2HG and L-2HG enantiomers. *N*-(p-toluenesulfonyl)-L-phenylalanyl chloride (TSPC) was used to derivatize 2HG. The formed diastereomers by TSPC labeling can efficiently improve the chromatographic separation of D-2HG and L-2HG. And derivatization by TSPC could also markedly increase the detection sensitivities by 291 and 346 folds for D-2HG and L-2HG, respectively. Using the developed method, we measured the contents of D-2HG and L-2HG in clear cell renal cell carcinoma (ccRCC) tissues. We observed 12.9 and 29.8 folds increase of D-2HG and L-2HG, respectively, in human ccRCC tissues compared to adjacent normal tissues. The developed chiral derivatization combined with LC-ESI-MS/MS analysis offers sensitive determination of D-2HG and L-2HG enantiomers, which benefits the precise diagnosis of 2HG related metabolic diseases.

It has long been known that cells undergoing growth exhibit changes in metabolism^1^. Accumulating evidences indicate that the contents of metabolites also can contribute to the control of gene expression and the alterations in metabolism are necessary for cell growth^2^. Cancer is a disease in which cells lose their normal checks on cell proliferation. In order to meet the increased requirements of proliferation, cancer cells often display fundamental changes in pathways of metabolism^3^.

One of the examples of the role of metabolism in cancer is the recently identified onco-metabolite with putative oncogenic property, 2-hydroxyglutarate (2HG), which is typically generated by mutated isocitrate dehydrogenase 1 and 2 (IDH1/2). Normal IDH1/2 catalyze the oxidative decarboxylation of isocitrate to generate α-ketoglutarate (α-KG). Because the mutations of IDH1/2 widely occur in gliomas, acute myeloid leukemia^4^,^5^, and other malignancies^6^,^7^, the content of 2HG therefore increases in these diseases. Due to the structural similarity between 2HG and α-KG, 2HG has been reported to be a competitor of α-KG and the elevated level of 2HG can inhibit multiple α-KG dependent dioxygenases, including approximate 30 histone demethylases and 3 TET (ten-eleven translocation) proteins that function on the epigenetic modifications of chromatin^8^. These data demonstrate that the IDH1/2 mutations result in production of the onco-metabolite 2HG, and excess 2HG that accumulates *in vivo* contributes to elevated risk of malignant tumors in patients due to the inhibition of α-KG dependent dioxygenases (Fig. 1). In this respect, quantification of the onco-metabolite 2HG may serve as an indicator of the formation and malignant progression of tumors and elucidate the in-depth mechanism between metabolism and gene expression.

2HG carries an asymmetric carbon atom in its carbon backbone and therefore occurs in two distinct forms, D-2-hydroxyglutarate (D-2HG) and L-2-hydroxyglutarate (L-2HG) (Fig. 1). It is important to note that both D-2HG and L-2HG are found in human body^9^. Although the enantiomers of D-2HG and L-2HG are identical in their physical and chemical properties, these metabolites are entirely different entities in term of their biochemical properties. Routine analytical methods to detect 2HG are not able to differentiate between D-2HG and L-2HG, and as a consequence the sum of the two metabolites is measured. Although the product caused by IDH1/2 mutations was demonstrated to be D-2HG^10^, numbers of reports measured total 2HG instead of D-2HG^11^,^12^,^13^,^14^. As a consequence, minor increase of D-2HG might be missed since endogenous levels of L-2HG in healthy individuals is equal to or exceeds the level of D-2HG. It is proposed that level of the IDH-specific D-2HG rather than total 2HG could increase the specificity to predict IDH1/2 mutations^9^,^15^. In addition, the accurate diagnosis of 2HG related metabolic diseases relies on the determination of the configuration of the enantiomers, either D-2HG or L-2HG in patients^16^,^17^. Therefore, determination of D-2HG and L-2HG instead of total 2HG is more persuasive and should be performed in clinical and scientific research.

Considering that D-2HG and L-2HG enantiomers have identical physical and chemical properties, separation of the enantiomers is challenging. Two strategies have been developed to separate and quantify D-2HG and L-2HG by utilizing chiral column^18^ or chiral derivatization^19^. Rashed *et al.*^18^ used a ristocetin A glycopeptide antibiotic silica gel bonded chiral column combined with mass spectrometry analysis to detect D-2HG and L-2HG. While this strategy required specific chiral column that is expensive. In addition, the detection sensitivity of the method is very low due to the poor ionization efficiency of 2HG in mass spectrometry. Chalmers *et al.*^19^ used chiral derivatization reagent of (D)-2-butanol and acetic anhydride to derivatize 2HG followed by gas chromatography/mass spectrometry (GC/MS) analysis. However, the two-step derivatization procedure was tedious and the derivatized D-2HG and L-2HG were still not well separated in subsequent GC/MS analysis.

Herein, we developed a novel strategy by chiral derivatization combined with liquid chromatography/tandem mass spectrometry (LC-ESI-MS/MS) analysis for highly sensitive determination of onco-metabolites D-2HG and L-2HG enantiomers. In this respect, *N*-(p-toluenesulfonyl)-L-phenylalanyl chloride (TSPC) was used for highly efficient labeling of D-2HG and L-2HG enantiomers under wild reaction conditions (Fig. 1). The results showed that the retention behavior of TSPC labeled D-2HG and L-2HG enantiomers was greatly improved and they can be well separated in the subsequent LC-ESI-MS/MS analysis. Moreover, TSPC chiral derivatization dramatically enhanced the detection sensitivities by 291 and 346 folds for D-2HG and L-2HG due to the introduction of easily ionizable group. Upon derivatization, the limits of detection (LODs) of D-2HG and L-2HG can reach 1.2 fmol and 1.0 fmol in solvent, respectively, which were, to our knowledge, the best detection sensitivities that can be achieved. Using this method, we simultaneously quantified onco-metabolites of D-2HG and L-2HG in human urines from patients with type 2 diabetes mellitus, lung cancer, colorectal cancer, nasopharyngeal carcinoma as well as in human clear cell renal cell carcinoma (ccRCC) tissues. We demonstrated that, compared to tumor adjacent normal tissues, the contents of D-2HG and L-2HG in ccRCC tissues significantly increased, suggesting that both D-2HG and L-2HG may serve as indicators in tumorgenesis.

## Results and Discussion

### Chiral Derivatization

**Figure 2:**
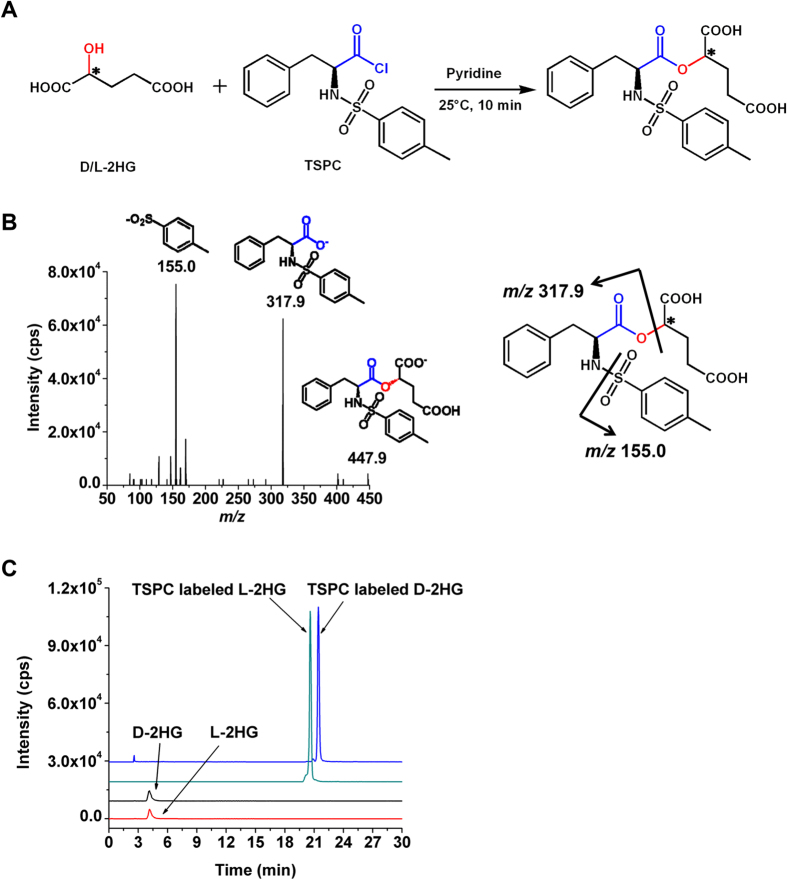
Characterization of TSPC derivatization. (**A**) Derivatization of D/L-2HG by TSPC. (**B**) Product ions spectrum of TSPC labeled L-2HG. (**C**) The extracted-ion chromatograms of D-2HG and L-2HG before and after TSPC labeling. The gradient elution method was used for the separation of TSPC labeled D-2HG and L-2HG.

2HG carries an asymmetric carbon atom and occurs in two distinct forms, D-2HG and L-2HG. To achieve good chromatographic separation of the enantiomers of D-2HG and L-2HG, here we employed TSPC that harbors a chiral carbon to derivatize D-2HG and L-2HG to form diastereomers (Fig. 2A), which may have slightly different interaction with stationary phase due to their different steric configuration and therefore offer the possibility for these diastereomers to be separated by LC.

It is known that acyl chloride moiety in TSPC has a high reaction activity with amino groups, alcohol and carboxyl groups. However, in this study hydroxyl group can be selectively derivatized by TSPC to form an ester using pyridine to neutralize the produced hydrochloric acid. The carboxyl group in 2HG will not react with TSPC under this reaction conditions and we did not detect any products formed through the reaction between the carboxyl group of 2HG and TSPC, which is due to the carboxyl group is a weaker nucleophile than hydroxyl. And anhydrous acetonitrile (ACN) was used as reaction solvent to prevent TSPC from hydrolyzing.

We then examined the TSPC labeled 2HG using LC-ESI-MS/MS. Figure 2B shows the fragmentation ions of TSPC labeled L-2HG, which clearly demonstrated the desired TSPC labeled 2HG was obtained. Moreover, the TSPC labeled 2HG was further confirmed by high-resolution mass spectrometry analysis (Figure S1, Supporting Information).

### Optimization of the Derivatization Conditions

To obtain good derivatization efficiency of 2HG, we optimized the derivatization conditions, including reaction time and temperature, and concentration of TSPC.

**Figure 3:**
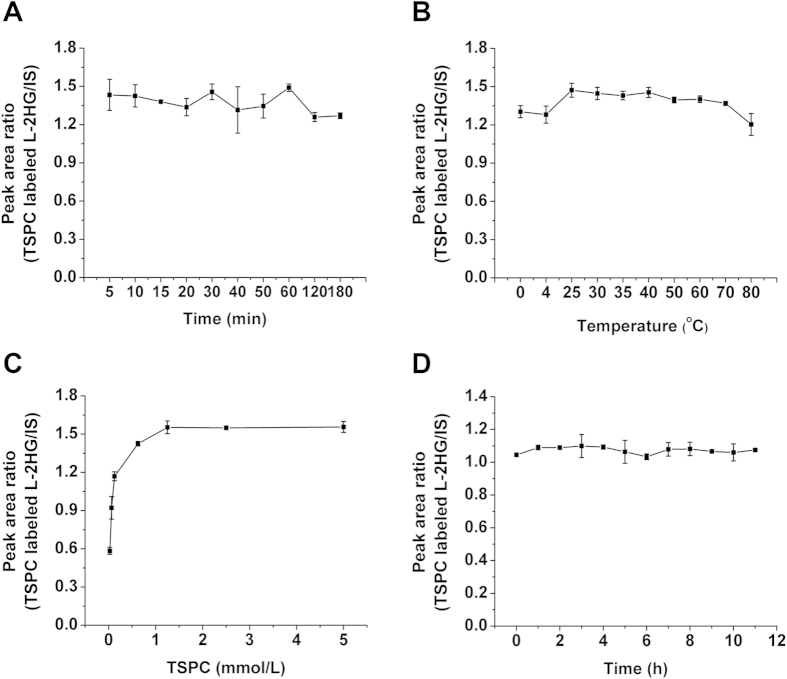
Optimization of derivatization conditions of L-2HG by TSPC. (**A**) Optimization of reaction time. (**B**) Optimization of reaction temperature. (**C**) Optimization of the concentration of TSPC. (**D**) Evaluation of the stability of TSPC labeled L-2HG. The isocratic elution method was used for the detection of L-2HG and TSPC labeled L-2HG.

We used L-2HG for the optimization of the derivatization conditions. We first optimized the reaction time, the result showed that the derivatization of L-2HG by TSPC was very fast and 5 min was enough for the efficient reaction (Fig. 3A). Therefore, 10 min was used as the reaction time to ensure the complete derivatization. We next optimized the reaction temperature. Our results demonstrated that the largest peak area ratio of TSPC labeled L-2HG versus internal standard (phthalic acid) can be achieved at 25 °C (Fig. 3B).

As for the optimization of the concentration of TSPC, the results showed that the peak area ratios of TSPC labeled L-2HG reach to the plateau when the concentration of TSPC is 1.25 mmol/L (Fig. 3C). Therefore, 1.25 mmol/L of TSPC was used for the following experiments. We finally evaluated the stability of the TSPC labeled L-2HG. The result demonstrated that the product was stable at least for 11 h (Fig. 3D).

Taken together, the optimized derivatization conditions for L-2HG by TSPC were at 25 °C for 10 min with the concentration of TSPC being 1.25 mmol/L. Under optimized derivatization conditions, more than 99% of L-2HG can react with TSPC to form the corresponding TSPC labeled L-2HG (data no shown), suggesting high derivatization efficiencies were achieved.

### Improvement of Chromatographic Separation and Detection Sensitivities of D-2HG and L-2HG upon TSPC Derivatization

The main purpose for derivatization is to improve the chromatographic separation as well as detection sensitivities of D-2HG and L-2HG enantiomers during LC-ESI-MS/MS analysis. The extracted-ion chromatogram shows that the retentions of D-2HG and L-2HG were relatively weak and they co-eluted on C18 column (Fig. 2C). However, the retentions of TSPC labeled D-2HG and L-2HG dramatically increased and can be well separated (Fig. 2C), suggesting that the formed diastereomers by TSPC labeling can efficiently improve the separation of D-2HG and L-2HG.

In addition to the improved chromatographic separation, derivatization by TSPC could also markedly increase the detection sensitivities by 291 and 346 folds for standard D-2HG and L-2HG dissolved in solvent, respectively, compared to their native forms (Table 1). The limits of detection (LODs) of TSPC labeled D-2HG and L-2HG were 1.2 and 1.0 fmol in solvent, respectively (Table 1). The remarkable increase in detection sensitivities achieved by TSPC derivatization can be attributed to several aspects. Firstly, the ionization of native 2HG is relatively poor under ESI-MS analysis; however, TSPC derivatization can effectively enhance the ionization efficiency, which therefore leads to increased mass spectrometry response. Secondly, the derivatization increased the retention of 2HG on reversed-phase chromatographic column, which results in longer retention time and thus elution within a higher ratio of organic solvent. Therefore the analytes could be ionized more effectively in ESI owing to higher spraying and desolvation efficiency under higher ACN content.

DATAN is a widely used reagent to derivatize enantiomers for their chiral separation^20^,^21^,^22^. In this respect, here we also employed DATAN to label D-2HG and L-2HG. The results showed that the separation resolution of DATAN labeled D-2HG and L-2HG was similar as that of TSPC labeled D-2HG and L-2HG (comparing Fig. 2C and Figure S2 in Supporting Information). However, the LODs of D-2HG and L-2HG upon DATAN derivatization were 115.0 fmol and 102.0 fmol in solvent, respectively, which are only about 3 folds lower than native 2HG and much higher than TSPC labeled D-2HG and L-2HG (Table 1). In addition, to achieve good derivatization efficiency, the required concentration of DATAN was high (250 mM), which may cause contamination of mass spectrometer. Therefore, derivatization of 2HG by TSPC offers much better detection sensitivities than by DATAN. In clinical research, sometimes the amount of clinical specimens is very limited, therefore TSPC derivatization combined LC-ESI-MS/MS analysis is more suitable for the sensitive determination of 2HG enantiomers with limited amount of clinical samples.

### Method Validation

The calibration curves of D-2HG and L-2HG were constructed in urine or tissue matrix by plotting the mean peak area ratios of TSPC labeled D-2HG and L-2HG to phthalic acid versus the corresponding D-2HG and L-2HG concentrations based on data obtained from triplicate measurements. The results showed that good linearities were obtained with the coefficient of determination (R^2^) being greater than 0.99 for both D-2HG and L-2HG in either urine or tissue matrix (Table S1, Supporting Information). In addition, the calculated LODs of D-2HG and L-2HG in urine matrix were 1.6 fmol and 1.5 fmol, respectively; and the LODs of D-2HG and L-2HG in tissue matrix were 1.9 fmol and 1.3 fmol, respectively.

The accuracy of the proposed method was assessed by comparing the contents of measured D-2HG and L-2HG spiked in urine samples to the theoretical D-2HG and L-2HG contents (Table 2). In addition, the repeatability of the developed method was evaluated by the measurement of intra- and inter-day precisions. The intra- and inter-day relative standard deviations (RSDs) were calculated at three different concentrations. Five parallel treatments of samples over a day gave the intra-day RSDs, and the inter-day RSDs were determined by treating samples independently for three consecutive days. The results showed that good accuracies were achieved, which is manifested by the relative errors (RE) ranging from −12.0% to 10.9% (Table 2). The results also showed that the intra- and inter-day RSDs were less than 10.7% and 12.1% for D-2HG and L-2HG, respectively (Table 2), demonstrating that good repeatability was achieved.

### Determination of D-2HG and L-2HG in Human Urine Samples

Utilizing this highly sensitive detection method, we successfully identified and quantified D-2HG and L-2HG in human urine samples from healthy controls (n = 20) and patients with type 2 diabetes mellitus (n = 20), lung cancer (n = 20), colorectal cancer (n = 20) and nasopharyngeal carcinoma (n = 20). Before analysis, we quantified creatinine in these samples according to previously described method^23^,^24^. The excretion of creatinine is rather constant over a long time and it is therefore used to normalize D-2HG and L-2HG contents in different samples in the current study, which is also a standard manner in the relatively quantitative analysis of urinary metabolite^25^,^26^.

**Figure 4:**
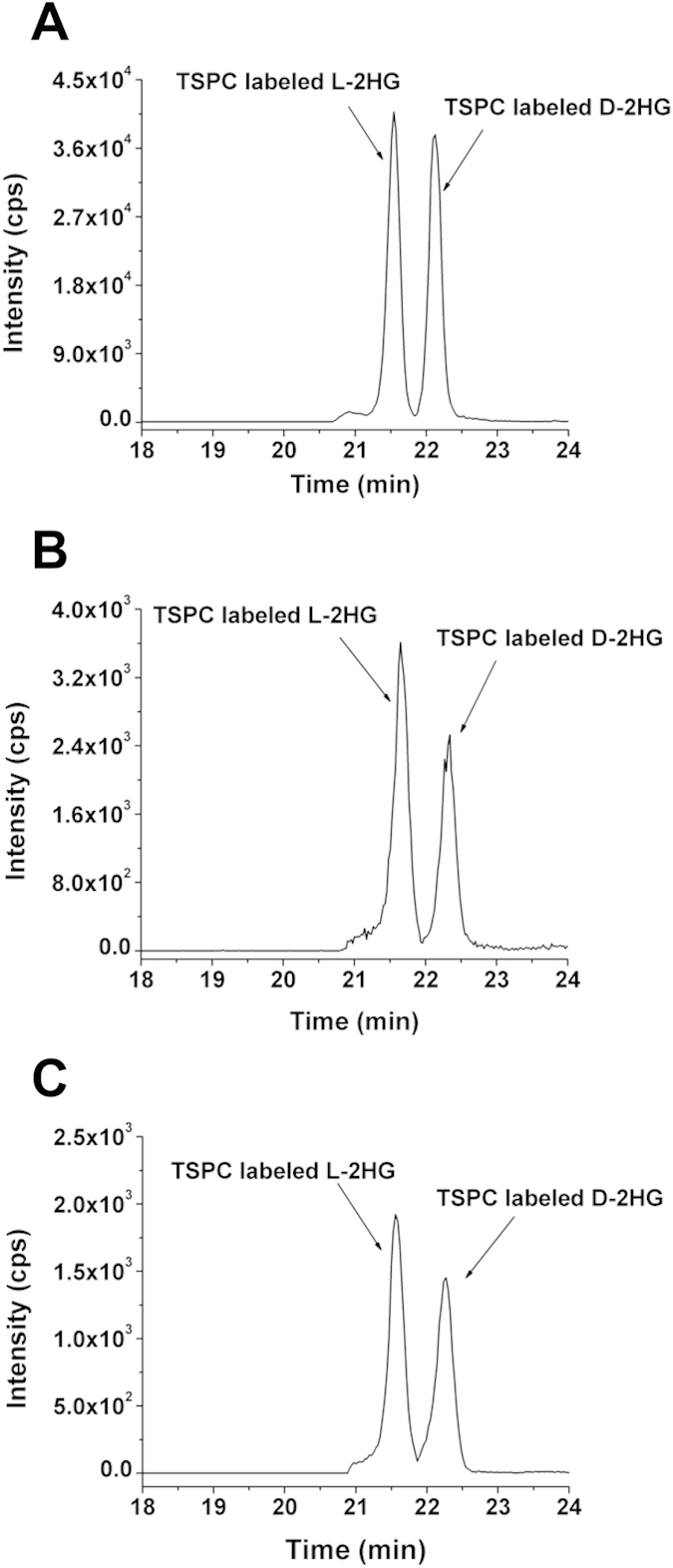
The extracted-ion chromatograms of TSPC labeled D-2HG and L-2HG. (**A**) TSPC labeled D-2HG and L-2HG standards. (**B**) TSPC labeled D-2HG and L-2HG detected in human urine sample. (**C**) TSPC labeled D-2HG and L-2HG detected in human ccRCC tissue sample.

**Figure 5:**
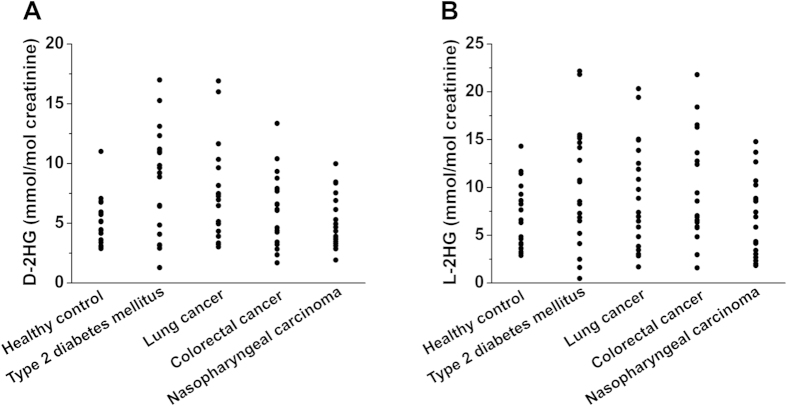
Measured contents of (A) D-2HG and (B) L-2HG in human urine samples from healthy controls (n = 20) and patients with type 2 diabetes mellitus (n = 20), lung cancer (n = 20), colorectal cancer (n = 20) and nasopharyngeal carcinoma (n = 20). Each point represents the measured content of 2HG in each urine sample.

Shown in Fig. 4A,B are the typical extracted-ion chromatograms of the TSPC labeled D-2HG and L-2HG standards and TSPC labeled D-2HG and L-2HG identified in human urine, respectively, suggesting D-2HG and L-2HG can be successfully detected in human urine. The quantitative results demonstrated that the contents of D-2HG and L-2HG in all urine samples ranged from 1.3–22.2 mmol/mol creatinine (Fig. 5, Table S2 in the Supporting Information). And no significant difference was observed between four kinds of patients and healthy controls (*p* > 0.05, t-test). These results indicated that the contents of D-2HG and L-2HG in human urines may not serve as efficient indicators of diabetes and cancers.

### Determination of D-2HG and L-2HG in ccRCC Tissue Samples

**Figure 6:**
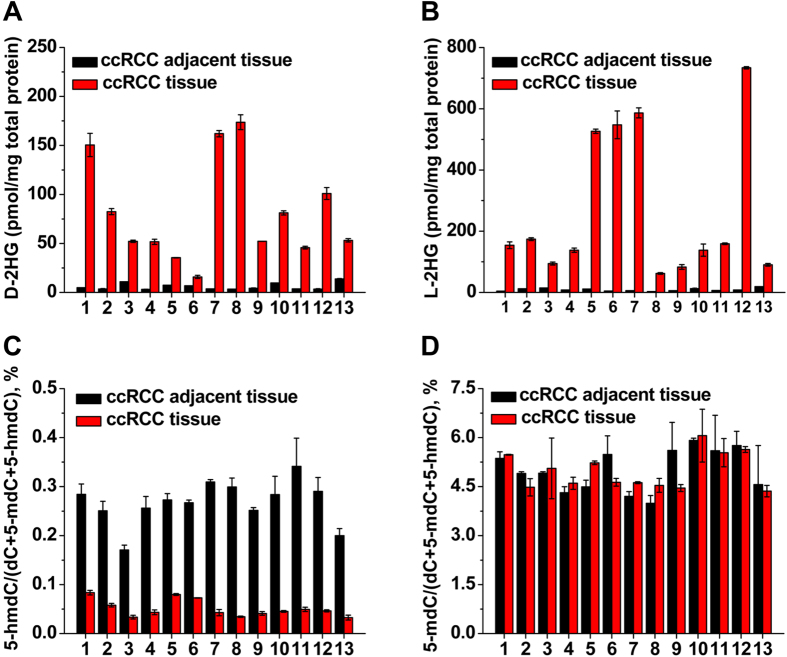
Measured contents of D-2HG and L-2HG, 5-mdC and 5-hmdC in paired ccRCC tissues and adjacent normal tissues (n = 13). (**A**) D-2HG contents in individual sample. (**B**) L-2HG contents in individual sample. (**C**) 5-hmdC contents in individual sample. (**D**) 5-mdC contents in individual sample.

We further quantified the amounts of D-2HG and L-2HG in 13 paired ccRCC tumor tissues and adjacent normal tissues. Shown in Fig. 4C is the typical extracted-ion chromatogram of TSPC labeled D-2HG and L-2HG detected in ccRCC tissue. Generally, we observed notable contents increase for both D-2HG and L-2HG in each ccRCC tissue compared to its own adjacent normal tissue (Fig. 6A,B). And the measured contents of D-2HG and L-2HG in ccRCC tissues and ccRCC adjacent normal tissues can be found in Table S3 in Supporting Information. The average contents of D-2HG in ccRCC tissues and ccRCC adjacent normal tissues were 77.5 and 6.0 pmol/mg total protein, respectively; and the average contents of L-2HG in ccRCC tissues and ccRCC adjacent normal tissues were 268.3 and 9.0 pmol/mg total protein, respectively (Table S3, Supporting Information). The results suggested significant increases of D-2HG and L-2HG in ccRCC tissues compared to adjacent normal tissues (12.9 folds for D-2HG, *p* = 6.9 × 10^−6^; 29.8 folds for L-2HG, *p* = 5.9 × 10^−4^, t-test).

Recent studies have demonstrated that 5-methycytosine (5-mdC) can be converted to 5-hydroxymethycytosine (5-hmdC) with α-KG as the cofactor, which constitutes the active DNA demethylation^27^,^28^,^29^. Because 2HG has been reported to be a competitor of α-KG^8^ and the elevated level of 2HG in ccRCC tissues therefore can inhibit α-KG dependent generation of 5-hmdC. In this respect, we examined the 5-hmdC contents in ccRCC tissues and adjacent normal tissues using our previously established method^30^ and the detailed quantification procedure can be found in Supporting Information. The results indeed showed the significant decrease of 5-hmdC in ccRCC tissues compared to adjacent normal tissues (Fig. 6C, *p* = 1.8 × 10^−14^). While, no obvious change of 5-mdC was observed between ccRCC tissues and adjacent normal tissues (Fig. 6D, *p* = 0.895). The decreased contents of 5-hmdC but not 5-mdC in multiple tumor tissues were also reported by our group and others^30^,^31^,^32^,^33^. These studies indicate that *in vivo* onco-metabolites of D-2HG and L-2HG have significant impact on epigenetic modifications, which therefore results in profound biological consequence. In this regard, determination of D-2HG and L-2HG could provide clues for the elucidation of tumorigenesis and pathogenesis of certain diseases. In addition, accurate detection of D-2HG and L-2HG enantiomers also can benefit the precise diagnosis of 2HG related metabolic diseases.

## Methods

### Chemicals and Reagents

L-2-hydroxyglutarate (L-2HG) was purchased from Sigma-Aldrich (St. Louis, MO, USA). D-2-hydroxyglutarate (D-2HG) and diacetyl-L-tartaric anhydride (DATAN) were purchased from J&K CHEMICA Company (Beijing, China). *N*-(p-toluenesulfonyl)-L-phenylalanyl chloride (TSPC) was purchased from TCI Co., Ltd. (Shanghai, China). Chromatographic grade methanol (MeOH) and acetonitrile (ACN) were purchased from TEDIA Co. Inc. (Ohio, USA). The tissue lysis buffer RIPA and Bradford Protein Assay Kit were bought from Beyotime Institute of Biotechnology (Shanghai, China). All other solvents and chemicals used were of analytical grade. Triethylamine (TEA), phthalic acid, formic acid, pyridine, acetic acid and dichloromethane (CH_2_Cl_2_) were purchased from Sinopharm Chemical Reagent Co., Ltd (Shanghai, China). The water used throughout the study was purified by a Milli-Q apparatus (Millipore, Bedford, MA).

The stock solutions of D-2HG, L-2HG and internal standard of phthalic acid were prepared in water at a concentration of 10 mM. The stock solution of TSPC was prepared in ACN at a concentration of 125 mM. The stock solution of DATAN was prepared in CH_2_Cl_2_ at a concentration of 250 mM. All stock solutions were stored at −20 °C.

As for the construction of calibration curves, diluted stock solutions of D-2HG, L-2HG and internal standard of phthalic acid were added to urine samples or 80% aqueous methanol-extracted homogenized tissues to obtain a series of concentrations of D-2HG and L-2HG at 0.01 to 10 μM in urine and 0.1 to 20 μM in tissues.

### Urine Samples

The urine samples from healthy controls (n = 20) and patients with type 2 diabetes mellitus (n = 20), lung cancer (n = 20), colorectal cancer (n = 20), nasopharyngeal carcinoma (n = 20) were collected from Hubei Cancer Hospital, China. All the patients were diagnosed with cancer for the first time and had not been given any treatment at the time point of urine samples collection. Informed consent was obtained from the study subjects, and an approval was granted by the Hubei Cancer Hospital Ethics Committee and met the declaration of Helsinki.

Pretreatment of urine samples was performed according to the previous reported procedure^34^,^35^. Briefly, the freshly collected urine samples were immediately centrifuged at 5000 × g for 10 min under 4 °C twice to remove insoluble debris. The supernatant was then filtered with a PRECLEANTM Syringe Filter Nylon membrane (13 mm × 0.22 μM, ANPEL Scientific Instrument Co., Shanghai). Finally, the supernatant was collected and stored at −80 °C.

Creatinine level in urine has been commonly used to normalize the urine metabolites^23^. Before analysis, the urinary creatinine in the urine samples was used for normalization of 2HG. Creatinine was determined according to the previously reported method with slight modification^35^. Briefly, the urine was diluted 50 folds by acetonitrile and then centrifuged at 5,000 × *g* for 10 min at 4 °C followed by analysis with HPLC-UV. The analysis was performed on the on a Phenomenex Luna NH_2_ column (150 mm × 4.6 mm, 5 μm) with acetonitrile/phosphate buffer solution (1.0 mM, pH 6.0) (85/15, V/V) as mobile phase at a flow rate of 1.0 mL/min. The column temperature was kept at 30 °C and the detection wavelength was set at 235 nm.

### Human Clear Cell Renal Cell Carcinoma (ccRCC) Tissues

A total of 26 clear cell renal cell carcinoma (ccRCC) tissue samples from 13 ccRCC patients, including 13 pairs of ccRCC tissues and matched tumor adjacent normal tissues without preoperative target therapy**/**chemotherapy were obtained from Department of Urology, Peking University First Hospital and Department of Urology, Peking University People’s Hospital. The patients with ccRCC did not have obvious metabolic diseases. And the pathological diagnosis of these specimens were reviewed and confirmed by two pathologists. Approvals for the study were obtained from the Ethical Committee of Peking University People’s Hospital. The ccRCC tissues and matched tumor adjacent normal tissues were kept at −80 °C. All the experiments were performed in accordance with Peking University People’s Hospital Ethics Committee’s guidelines and regulations.

Extraction of 2HG from tissue samples was performed according to the preciously described method^36^. Briefly, 5–15 mg tissue was weighed and added to 1 mL 80% aqueous methanol (pre-chilled to −80 °C) in a glass pestle. The tissue was grounded manually for 10 min on ice and then transferred to a tube followed with centrifugation at 14000 × *g* for 10 min under 4 °C. Finally, the supernatant was collected and subjected to subsequent analysis.

The measured contents of D-2HG and L-2HG were normalized to total protein content. The ccRCC tissues lysate was generated using RIPA buffer according to previously described procedure^37^. And protein contents were measured by standard Branford protein assay.

### Chiral Derivatization

TSPC was used to derivatize 2-HG followed with LC-ESI-MS/MS analysis. The chiral derivatization reaction conditions, including reaction time and temperature, concentration of TSPC, were optimized to achieve the best derivatization efficiency.

We first optimized the reaction time. Generally, L-2HG (2 nmol) was dried with nitrogen gas at 37 °C followed with adding 160 μL TSPC (2.5 mM in ACN) and 2 μL pyridine. The mixture was then incubated at 40 °C for different times ranging from 5 min to 3 h with shaking at 1,500 r/min. The derivatization temperature was optimized ranging from 0 °C to 80 °C, and the reactions were performed for 10 min with the concentration of TSPC being 1.25 mmol/L. The concentration of TSPC was also optimized ranging from 0.025 to 5 mmol/L. After the derivatization reaction, the mixture was dried with nitrogen gas at 37 °C and then redissolved in 100 μL 50% aqueous ACN. Finally, 10 μL phthalic acid (1 mmol/L) was added as the internal standard to correct the signal fluctuation during mass spectrometry analysis.

All the optimization experiments were performed on a HPLC-ESI-MS system consisting of a Shimadzu LC-20AD HPLC system (Tokyo, Japan) and a Shimadzu LCMS-2010EV mass spectrometer (Tokyo, Japan). Data acquisition and processing were performed using LCSolutions 5.42 SP4 software (Tokyo, Japan). Drying and nebulizer gases of nitrogen were set at 15 L/min and 1.5 L/min, respectively. Capillary voltage was 4.5 kV. Curved desolvation line and heat block temperatures were set at 250 °C and 200 °C, respectively. The detector voltage was set at 1.05 V. A Shimadzu VP-ODS column (150 mm × 2.1 mm i.d., 5 μm, Tokyo, Japan) was used for the separation. The column temperature was set at 35 °C. The isocratic elution consisted of acetonitrile/water (50:50, V/V) with 0.1% (V/V) formic acid at a flow rate of 0.2 mL/min. The negative selected ion monitoring (SIM) mode was used.

For comparison, the derivatization of D-2HG and L-2HG with diacetyl-L-tartaric anhydride (DATAN) was also performed and the detailed procedure can be found in Supporting Information.

### Determination of D-2HG and L-2HG in Human Urine and Tissue Samples

The diluted urine (50 times) or supernatant extracted from tissue samples was dried with nitrogen gas at 37 °C. Then 160 μL TSPC (2.5 mM in ACN) and 2 μL pyridine were added for the chiral derivatization under optimized conditions. After the reaction, the mixture was dried under nitrogen gas at 37 °C and then redissolved in 100 μL 50% aqueous ACN containing phthalic acid. And 10 μL of the sample was subjected to LC-ESI-MS/MS analysis.

The quantification of TSPC labeled D-2HG and L-2HG were performed on the LC-ESI-MS/MS system consisting of an AB 3200 QTRAP mass spectrometer (Applied Biosystems, Foster City, CA, USA) with an electrospray ionization source (Turbo Ionspray) and a Shimadzu LC-20AD HPLC (Tokyo, Japan) with two LC-20AD pumps, a SIL-20A auto sampler, a CTO-20AC thermostated column compartment and a DGU-20A3 degasser. Data acquisition and processing were performed using AB SCIEX Analyst 1.5 Software (Applied Biosystems, Foster City, CA, USA). The HPLC separation was performed on an Inertsil ODS-3 column (250 mm × 2.0 mm i.d., 5 μm, Tokyo, Japan) at 35 °C. Formic acid in water (0.1%, v/v, solvent A) and a mixture of ACN and MeOH (50:50, V/V, solvent B) were employed as the mobile phase. A gradient of 3 min 30% B, 7 min 30–70% B, 15 min 70% B, 1 min 70–30% B, and 14 min 30% B was used. The flow rate of mobile phase was set at 0.2 mL/min.

The mass spectrometry detection was performed using multiple reaction monitoring (MRM) under negative ion mode. The mass transitions (precursor ions → product ions) were 447.9 → 317.9 and 447.9 → 155.0 for TSPC labeled 2HG, 146.9 → 128.9 for 2HG, and 164.9 → 120.9 for internal standard of phthalic acid. The MRM parameters of all analytes were optimized to achieve maximal detection sensitivity.

High resolution mass spectrometry experiments were performed on the QTOF-MS system consisting of a MicrOTOF-Q orthogonal-accelerated TOF mass spectrometer (Bruker Daltonics, Bremen, Germany) with an ESI source (Turbo Ionspray). Data acquisition and processing were performed using Bruker Daltonics Control 3.4 and Bruker Daltonics Data analysis 4.0 software.

### Statistical Analysis

The statistical data were processed with SPSS 19.0 software (SPSS Inc.). The paired t-test was performed to evaluate the differences of D-2HG and L-2HG in urine and ccRCC tissue samples. All *p* values were two-sided, and *p* values < 0.05 were considered to have statistical significance.

## Additional Information

**How to cite this article**: Cheng, Q.-Y. *et al.* Sensitive Determination of Onco-metabolites of D- and L-2-hydroxyglutarate Enantiomers by Chiral Derivatization Combined with Liquid Chromatography/Mass Spectrometry Analysis. *Sci. Rep.* **5**, 15217; doi: 10.1038/srep15217 (2015).

## Supplementary Material

Supporting Information

## Figures and Tables

**Figure 1:**
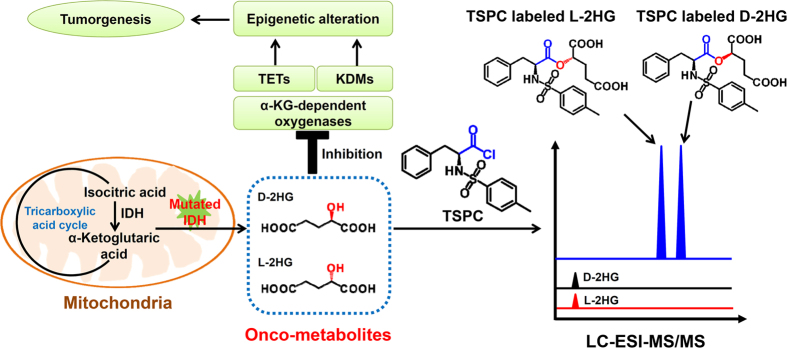
Schematic diagram for the generation of 2HG by mutated IDHs and determination of D-2HG and L-2HG by TSPC derivatization strategy. TETs, ten-eleven translocation; KDMs, histone lysine demethylases.

**Table 1:** LODs of D-2HG and L-2HG obtained by different methods.

Analytical methods	LODs (fmol)		Increased folds of the detection sensitivity	
	D-2HG	L-2HG	D-2HG	L-2HG
Underivazitation	348.6	345.6	–	–
DATAN labeling	115.0	102.0	3.0	3.4
TSPC labeling	1.2	1.0	291	346

Derivatizations were performed under their own optimized conditions. The LODs were obtained with analytes dissolved in solvent of 50% aqueous ACN.

**Table 2:** Accuracy and precision (intra- and inter-day) for the determination of D-2HG and L-2HG spiked in urine samples by TSPC chiral derivatization combined LC-ESI-MS/MS analysis.

Analytes	Theoretical value (pmol)	Measured value (pmol)	Relative error (%)	Intra-day (RSD%, n = 5)	Inter-day (RSD%, n = 3)
D-2HG	0.02	0.02	2.0	3.0	2.7
	2.00	2.19	9.4	1.9	10.7
	50.00	44.00	−12.0	1.4	8.6
L-2HG	0.02	0.02	0.9	3.5	12.1
	2.00	2.22	10.9	2.2	1.9
	50.00	47.00	−6.1	1.8	2.8
