# Supplementary material for: Sensitive Determination of Onco-metabolites of D- and L-2-hydroxyglutarate Enantiomers by Chiral Derivatization Combined with Liquid Chromatography/Mass Spectrometry Analysis
Source: Sci Rep. 2015 Oct 13;5:15217. doi: 10.1038/srep15217 (PMC4602309; doi:10.1038/srep15217)
Supplement: Supporting Information [file srep15217-s1.doc]

Supporting Information

for

**Sensitive Determination of Onco-metabolites of D- and L-2-hydroxyglutarate Enantiomers by Chiral Derivatization Combined with Liquid Chromatography/Mass Spectrometry Analysis**

Qing-Yun Cheng,^1,†^ Jun Xiong,^1,†^ Wei Huang,^1^ Qin Ma,^2^ Weimin Ci,^2^ Yu-Qi Feng,^1^,* Bi-Feng Yuan^1^,*

^1^ Key Laboratory of Analytical Chemistry for Biology and Medicine (Ministry of Education), Department of Chemistry, Wuhan University, Wuhan 430072, China.

^2^ Key Laboratory of Genomic and Precision Medicine, Beijing Institute of Genomics, Chinese Academy of Sciences, Beijing, 100101, China.

† These authors contributed equally to this work.

* To whom correspondence should be addressed. Tel. +86-27-68755595; fax. +86-27-68755595. E-mail address: [bfyuan@whu.edu.cn](mailto:bfyuan@whu.edu.cn); yqfeng@whu.edu.cn.

**Analysis of D-2HG and L-2HG with diacetyl-L-tartaric anhydride (DATAN) derivatization**

A 20-μL 2-HG (0.1 mM) aliquot was dried with nitrogen gas at 35°C and then 50 μL DATAN (250 mM, dissolved in dichloromethane/acetic acid, 4:1, v/v) was added. The reaction was performed at 75°C for 30 min with shaking at 1,500 r/min. The mixture was dried by nitrogen gas at room temperature. The residue was then redissolved in 100 μL of water and 10 μL was subjected to LC-ESI-MS/MS analysis.

Analysis of DATAN labeled D-2HG and L-2HG was performed on the LC-ESI-MS/MS system consisting of an AB 3200 QTRAP mass spectrometer (Applied Biosystems, Foster City, CA). Data acquisition and processing were performed using AB SCIEX Analyst 1.5 Software (Applied Biosystems, Foster City, CA). The HPLC separation was performed on an Inertsil ODS-3 column (250 mm × 2.0 mm i.d., 5 μm, Tokyo, Japan). The isocratic elution consisted of 5 mmol/L ammonium formate in water/acetonitrile (95/5, v/v) at a flow rate of 0.2 mL/min.

The mass spectrometry detection was performed under negative electrospray ionization mode. The target analytes were monitored by multiple reaction monitoring (MRM) using the mass transitions (precursor ions → product ions) of *m/z* 362.7 → 146.8 for DATAN-labeled 2-HG, 146.9 → 128.9 for 2-HG. The MRM parameters of all analytes were optimized to achieve maximal detection sensitivity.

**Analysis of 5-methyltytosine (5-mdC) and 5-hydroxymethylcytosine (5-hmdC) contents in genomic DNA of ccRCC tissues**

ccRCC tissues DNA were extracted using E.Z.N.A.® DNA Kit (Omega Bio-Tek Inc., Norcross, GA) according to the manufacturer’s recommended procedure. The concentration of the puriﬁed genomic DNA was determined on a B-500 spectrophotometer (Metash Instruments Co., Ltd., Shanghai, China).

As for enzymatic digestion, genomic DNA (1 μg in 16 μL H_2_O) was first denatured by heating at 95ºC for 5 min and then chilling on ice for 2 min. After adding 1/10 volume (2 μL) of S1 nuclease buffer (30 mM CH_3_COONa, pH 4.6, 280 mM NaCl, 1 mM ZnSO_4_) and 360 units (2 μL) of S1 nuclease, the mixture (20 μL) was then incubated at 37 ºC for 4 h. To the resulting solution was subsequently added 10 μL of alkaline phosphatase buffer (50 mM Tris-HCl, 10 mM MgCl_2_, pH 9.0), 0.01 units (5 μL) of venom phosphodiesterase I, 30 units (1 μL) of alkaline phosphatase and 64 μL H_2_O. Then, the incubation was continued at 37ºC for an additional 2 h followed by extraction with equal volume of chloroform twice. The resulting aqueous layer was collected and lyophilized to dryness and reconstituted in 100 μL water. After that, 30 μL of the obtained samples were subjected to LC-ESI-MS/MS analysis.

Analysis of nucleosides was performed on the LC-ESI-MS/MS system consisting of an AB 3200 QTRAP mass spectrometer (Applied Biosystems, Foster City, CA, USA). Data acquisition and processing were performed using AB SCIEX Analyst 1.5 Software (Applied Biosystems, Foster City, CA). The HPLC separation was performed on a HiSep C18-T column (150 mm×2.1 mm i.d., 5 μm, Weltech Co., Ltd., Wuhan, China) with a flow rate of 0.2 mL/min at 35°C. Formic acid in water (0.1%, v/v, solvent A) and formic acid in methanol (0.1%, v/v, solvent B) were employed as mobile phase. A gradient of 5 min 5% B, 10 min 5-30% B, 5 min 30-50% B, 3 min 50% B-5% B and 17 min 5% B was used.

The mass spectrometry detection was performed under positive electrospray ionization mode. The target nucleosides were monitored by multiple reaction monitoring (MRM) using the mass transitions (precursor ions → product ions) of dA (252.4 → 136.2), dG (268.4 → 152.1), dC (228.4 → 112.2), T (243.3 → 127.2), 5-mdC (242.3 → 126.1), 5-hmdC (258.2 → 142.1). The MRM parameters of all analytes were optimized to achieve maximal detection sensitivity.

The contents of 5-mdC and 5-hmdC were calculated using the following expression:


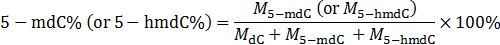


where *M*_5-mdC_ is the molar quantity of 5-mdC, *M*_5-hmdC_ is the molar quantity of 5-hmdC, and *M*_dC_ is the molar quantity of dC determined in DNA.

Table S1. Linearities of D-2HG and L-2HG by TSPC derivatization combined with LC-ESI-MS/MS analysis in urine or tissue matrix.

| Matrix | Analysts | Linear range (pmol) | Linear regression | R^2^ |
| --- | --- | --- | --- | --- |
| Urine | D-2HG | 0.1 - 100 | y = 0.9509 x - 0.2798 | 0.9990 |
|  | L-2HG | 0.1 - 100 | y = 0.9743 x - 0.1152 | 0.9986 |
| Tissue | D-2HG | 1 - 200 | y = 2.9820 x + 1.1913 | 0.9966 |
|  | L-2HG | 1 - 200 | y = 3.0842 x + 1.6284 | 0.9984 |

Table S2. Measured contents of D-2HG and L-2HG in human urine samples from healthy controls (n=20) and patients with type 2 diabetes mellitus (n=20), lung cancer (n=20), colorectal cancer (n=20) and nasopharyngeal carcinoma (n=20).

| No. | Category | D-2HG/Creatinine (mmol/mol) | L-2HG/Creatinine (mmol/mol) |
| --- | --- | --- | --- |
| 1 | Healthy control | 5.9 ± 0.04 | 6.3 ± 0.1 |
| 2 | Healthy control | 3.4 ± 0.1 | 4.1 ± 0.1 |
| 3 | Healthy control | 5.2 ± 0.03 | 4.6 ± 0.04 |
| 4 | Healthy control | 6.8 ± 0.1 | 3.6 ± 0.1 |
| 5 | Healthy control | 2.9 ± 0.1 | 3.1 ± 0.03 |
| 6 | Healthy control | 3.0 ± 0.04 | 4.0 ± 0.01 |
| 7 | Healthy control | 4.2 ± 0.05 | 3.6 ± 0.1 |
| 8 | Healthy control | 6.8 ± 0.3 | 3.3 ± 0.1 |
| 9 | Healthy control | 3.6 ± 0.02 | 2.9 ± 0.03 |
| 10 | Healthy control | 5.1 ± 0.03 | 4.8 ± 0.4 |
| 11 | Healthy control | 5.7 ± 0.1 | 10.1 ± 0.5 |
| 12 | Healthy control | 11.0 ± 0.2 | 14.3 ± 0.4 |
| 13 | Healthy control | 3.6 ± 0.07 | 8.3 ± 0.08 |
| 14 | Healthy control | 3.1 ± 0.07 | 6.6 ± 0.02 |
| 15 | Healthy control | 4.4 ± 0.2 | 8.6 ± 0.5 |
| 16 | Healthy control | 4.5 ± 0.1 | 8.6 ± 0.2 |
| 17 | Healthy control | 6.8 ± 0.05 | 11.5 ± 0.4 |
| 18 | Healthy control | 7.1 ± 0.3 | 11.7 ± 0.8 |
| 19 | Healthy control | 3.0 ± 0.2 | 7.6 ± 0.08 |
| 20 | Healthy control | 5.8 ± 0.2 | 9.3 ± 0.3 |
| 1 | Type 2 diabetes mellitus | 17.0 ± 0.1 | 10.7 ± 0.1 |
| 2 | Type 2 diabetes mellitus | 1.3 ± 0.01 | 1.8 ± 0.06 |
| 3 | Type 2 diabetes mellitus | 13.1 ± 0.1 | 8.5 ± 0.03 |
| 4 | Type 2 diabetes mellitus | 9.2 ± 0.1 | 7.3 ± 0.1 |
| 5 | Type 2 diabetes mellitus | 3.1 ± 0.04 | 1.6 ± 0.03 |
| 6 | Type 2 diabetes mellitus | 11.2 ± 0.1 | 6.8 ± 0.1 |
| 7 | Type 2 diabetes mellitus | 6.4 ± 0.05 | 4.1 ± 0.05 |
| 8 | Type 2 diabetes mellitus | 3.2 ± 0.1 | 2.5 ± 0.3 |
| 9 | Type 2 diabetes mellitus | 9.8 ± 0.03 | 6.5 ± 0.7 |
| 10 | Type 2 diabetes mellitus | 9.7 ± 0.2 | 15.3 ± 1.4 |
| 11 | Type 2 diabetes mellitus | 2.9 ± 0.03 | 5.2 ± 0.5 |
| 12 | Type 2 diabetes mellitus | 4.1 ± 0.05 | 8.3 ± 0.3 |
| 13 | Type 2 diabetes mellitus | 15.3 ± 0.3 | 21.8 ± 0.2 |
| 14 | Type 2 diabetes mellitus | 6.5 ± 0.08 | 10.6 ± 0.2 |
| 15 | Type 2 diabetes mellitus | 12.3 ± 0.3 | 22.2 ± 0.4 |
| 16 | Type 2 diabetes mellitus | 11.0 ± 0.2 | 14.2 ± 0.5 |
| 17 | Type 2 diabetes mellitus | 4.8 ± 0.07 | 15.5 ± 0.8 |
| 18 | Type 2 diabetes mellitus | 11.2 ± 0.3 | 14.7 ± 0.2 |
| 19 | Type 2 diabetes mellitus | 10.9 ± 0.1 | 15.2 ± 0.2 |
| 20 | Type 2 diabetes mellitus | 8.9 ± 0.3 | 12.8 ± 0.7 |
| 1 | Lung cancer | 4.9 ± 0.02 | 2.8 ± 0.1 |
| 2 | Lung cancer | 7.5 ± 0.05 | 7.4 ± 0.2 |
| 3 | Lung cancer | 3.9 ± 0.03 | 3.0 ± 0.1 |
| 4 | Lung cancer | 8.2 ± 0.02 | 6.9 ± 0.1 |
| 5 | Lung cancer | 3.2 ± 0.04 | 3.5 ± 0.2 |
| 6 | Lung cancer | 16.9 ± 0.02 | 12.5 ± 0.7 |
| 7 | Lung cancer | 10.3 ± 0.01 | 3.8 ± 0.06 |
| 8 | Lung cancer | 16.0 ± 0.1 | 6.5 ± 0.01 |
| 9 | Lung cancer | 5.0 ± 0.02 | 4.8 ± 0.10 |
| 10 | Lung cancer | 3.3 ± 0.03 | 17.0 ± 0.05 |
| 11 | Lung cancer | 3.0 ± 0.08 | 5.9 ± 0.3 |
| 12 | Lung cancer | 6.5 ± 0.3 | 10.8 ± 0.3 |
| 13 | Lung cancer | 7.0 ± 0.2 | 15.0 ± 0.9 |
| 14 | Lung cancer | 5.1 ± 0.08 | 9.8 ± 0.9 |
| 15 | Lung cancer | 9.7 ± 0.2 | 19.4 ± 0.3 |
| 16 | Lung cancer | 7.3 ± 0.1 | 15.1 ± 0.6 |
| 17 | Lung cancer | 7.3 ± 0.8 | 11.9 ± 0.4 |
| 18 | Lung cancer | 11.7 ± 0.5 | 20.3 ± 0.7 |
| 19 | Lung cancer | 7.4 ± 0.3 | 13.9 ± 0.9 |
| 20 | Lung cancer | 4.3 ± 0.2 | 8.9 ± 0.2 |
| 1 | Colorectal cancer | 13.3 ± 0.2 | 12.4 ± 0.5 |
| 2 | Colorectal cancer | 4.6 ± 0.01 | 1.6 ± 0.03 |
| 3 | Colorectal cancer | 4.6 ± 0.05 | 2.9 ± 0.1 |
| 4 | Colorectal cancer | 7.7 ± 0.1 | 6.9 ± 0.07 |
| 5 | Colorectal cancer | 7.8 ± 0.07 | 5.9 ± 0.06 |
| 6 | Colorectal cancer | 6.6 ± 0.1 | 5.8 ± 0.2 |
| 7 | Colorectal cancer | 1.7 ± 0.01 | 1.6 ± 0.04 |
| 8 | Colorectal cancer | 6.6 ± 0.1 | 4.8 ± 0.02 |
| 9 | Colorectal cancer | 2.8 ± 0.2 | 6.5 ± 0.2 |
| 10 | Colorectal cancer | 8.8 ± 0.5 | 16.3 ± 0.5 |
| 11 | Colorectal cancer | 4.4 ± 0.1 | 13.6 ± 0.6 |
| 12 | Colorectal cancer | 3.4 ± 0.1 | 6.6 ± 0.2 |
| 13 | Colorectal cancer | 6.2 ± 0.3 | 16.5 ± 0.6 |
| 14 | Colorectal cancer | 2.4 ± 0.1 | 6.4 ± 0.09 |
| 15 | Colorectal cancer | 9.3 ± 0.2 | 21.8 ± 0.7 |
| 16 | Colorectal cancer | 4.3 ± 0.1 | 7.0 ± 0.4 |
| 17 | Colorectal cancer | 7.9 ± 0.2 | 12.7 ± 1.3 |
| 18 | Colorectal cancer | 6.0 ± 0.1 | 9.4 ± 0.3 |
| 19 | Colorectal cancer | 3.2 ± 0.05 | 8.6 ± 0.5 |
| 20 | Colorectal cancer | 10.4 ± 0.2 | 18.4 ± 0.6 |
| 1 | Nasopharyngeal carcinoma | 4.4 ± 0.04 | 2.0 ± 0.03 |
| 2 | Nasopharyngeal carcinoma | 3.5 ± 0.00 | 1.8 ± 0.03 |
| 3 | Nasopharyngeal carcinoma | 3.1 ± 0.05 | 4.2 ± 0.05 |
| 4 | Nasopharyngeal carcinoma | 11.0 ± 0.1 | 10.7 ± 0.1 |
| 5 | Nasopharyngeal carcinoma | 6.1 ± 0.1 | 3.0 ± 0.07 |
| 6 | Nasopharyngeal carcinoma | 2.9 ± 0.02 | 2.7 ± 0.01 |
| 7 | Nasopharyngeal carcinoma | 3.5 ± 0.01 | 2.3 ± 0.01 |
| 8 | Nasopharyngeal carcinoma | 1.9 ± 0.02 | 3.4 ± 0.1 |
| 9 | Nasopharyngeal carcinoma | 7.5 ± 0.02 | 4.2 ± 0.03 |
| 10 | Nasopharyngeal carcinoma | 8.5 ± 0.7 | 8.8 ± 0.3 |
| 11 | Nasopharyngeal carcinoma | 5.3 ± 0.2 | 12.7 ± 0.2 |
| 12 | Nasopharyngeal carcinoma | 3.9 ± 0.02 | 10.2 ± 0.5 |
| 13 | Nasopharyngeal carcinoma | 5.0 ± 0.2 | 7.4 ± 0.08 |
| 14 | Nasopharyngeal carcinoma | 2.9 ± 0.1 | 5.9 ± 0.1 |
| 15 | Nasopharyngeal carcinoma | 4.7 ± 0.06 | 8.6 ± 0.4 |
| 16 | Nasopharyngeal carcinoma | 3.8 ± 0.02 | 8.7 ± 0.04 |
| 17 | Nasopharyngeal carcinoma | 6.9 ± 0.5 | 13.7 ± 0.02 |
| 18 | Nasopharyngeal carcinoma | 8.4 ± 0.1 | 14.8 ± 0.02 |
| 19 | Nasopharyngeal carcinoma | 3.7 ± 0.3 | 6.9 ± 0.5 |
| 20 | Nasopharyngeal carcinoma | 3.3 ± 0.1 | 4.3 ± 0.04 |

Table S3. Measured contents of D-2HG and L-2HG in paired ccRCC tissues and adjacent normal tissues.

| No. | Category | D-2HG (pmol)/mg protein | L-2HG (pmol)/mg protein |
| --- | --- | --- | --- |
| 1 | ccRCC adjacent normal tissue | 5.0 ± 0.3 | 4.2 ± 0.3 |
|  | ccRCC tissue | 150.5 ± 11.9 | 154.2 ± 10.8 |
| 2 | ccRCC adjacent normal tissue | 3.5 ± 0.9 | 12.1 ± 0.6 |
|  | ccRCC tissue | 82.4 ± 3.2 | 174.1 ± 4.7 |
| 3 | ccRCC adjacent normal tissue | 10.9 ± 0.2 | 14.7 ± 0.3 |
|  | ccRCC tissue | 52.2 ± 1.2 | 94.3 ± 4.8 |
| 4 | ccRCC adjacent normal tissue | 3.1 ± 0.6 | 8.4 ± 0.5 |
|  | ccRCC tissue | 51.7 ± 2.6 | 137.8 ± 7.0 |
| 5 | ccRCC adjacent normal tissue | 7.4 ± 0.4 | 11.4 ± 0.2 |
|  | ccRCC tissue | 35.4 ± 0.2 | 526.5 ± 6.9 |
| 6 | ccRCC adjacent normal tissue | 6.8 ± 0.2 | 4.8 ± 0.2 |
|  | ccRCC tissue | 15.9 ± 1.6 | 547.4 ± 45.4 |
| 7 | ccRCC adjacent normal tissue | 3.6 ± 0.4 | 6.2 ± 0.1 |
|  | ccRCC tissue | 162.0 ± 3.3 | 586.4 ± 16.3 |
| 8 | ccRCC adjacent normal tissue | 3.5 ± 0.0 | 3.1 ± 0.2 |
|  | ccRCC tissue | 173.7 ± 7.6 | 62.0 ± 2.6 |
| 9 | ccRCC adjacent normal tissue | 4.3 ± 0.9 | 6.2 ± 0.0 |
|  | ccRCC tissue | 52.2 ± 0.1 | 83.2 ± 7.6 |
| 10 | ccRCC adjacent normal tissue | 9.7 ± 0.4 | 12.5 ± 3.0 |
|  | ccRCC tissue | 81.2 ± 2.1 | 138.0 ± 19.9 |
| 11 | ccRCC adjacent normal tissue | 3.7 ± 0.1 | 6.4 ± 0.2 |
|  | ccRCC tissue | 45.8 ± 1.3 | 159.0 ± 2.5 |
| 12 | ccRCC adjacent normal tissue | 3.4 ± 0.8 | 8.1 ± 0.2 |
|  | ccRCC tissue | 100.9 ± 6.1 | 733.8 ± 33.9 |
| 13 | ccRCC adjacent normal tissue | 13.8 ± 0.8 | 19.5 ± 1.1 |
|  | ccRCC tissue | 53.2 ± 1.8 | 90.5 ± 0.5 |
| Mean content in ccRCC adjacent normal tissues | | 6.0 ± 3.4 | 9.0 ± 4.7 |
| Mean content in ccRCC tissues | | 77.5 ± 50.8 | 268.3 ± 236.1 |


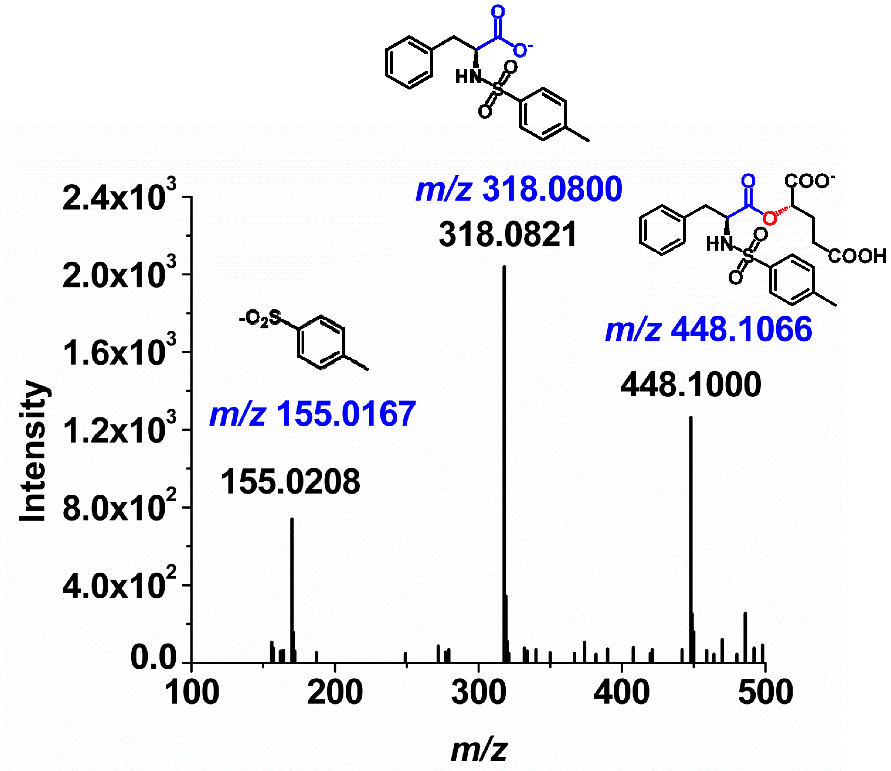


**Figure S1.** The product ion spectrum of TSPC labeled L-2HG standard obtained by high-resolution mass spectrometry. Highlight in blue are the theoretical molecular weights of TSPC labeled L-2HG and its product ions.


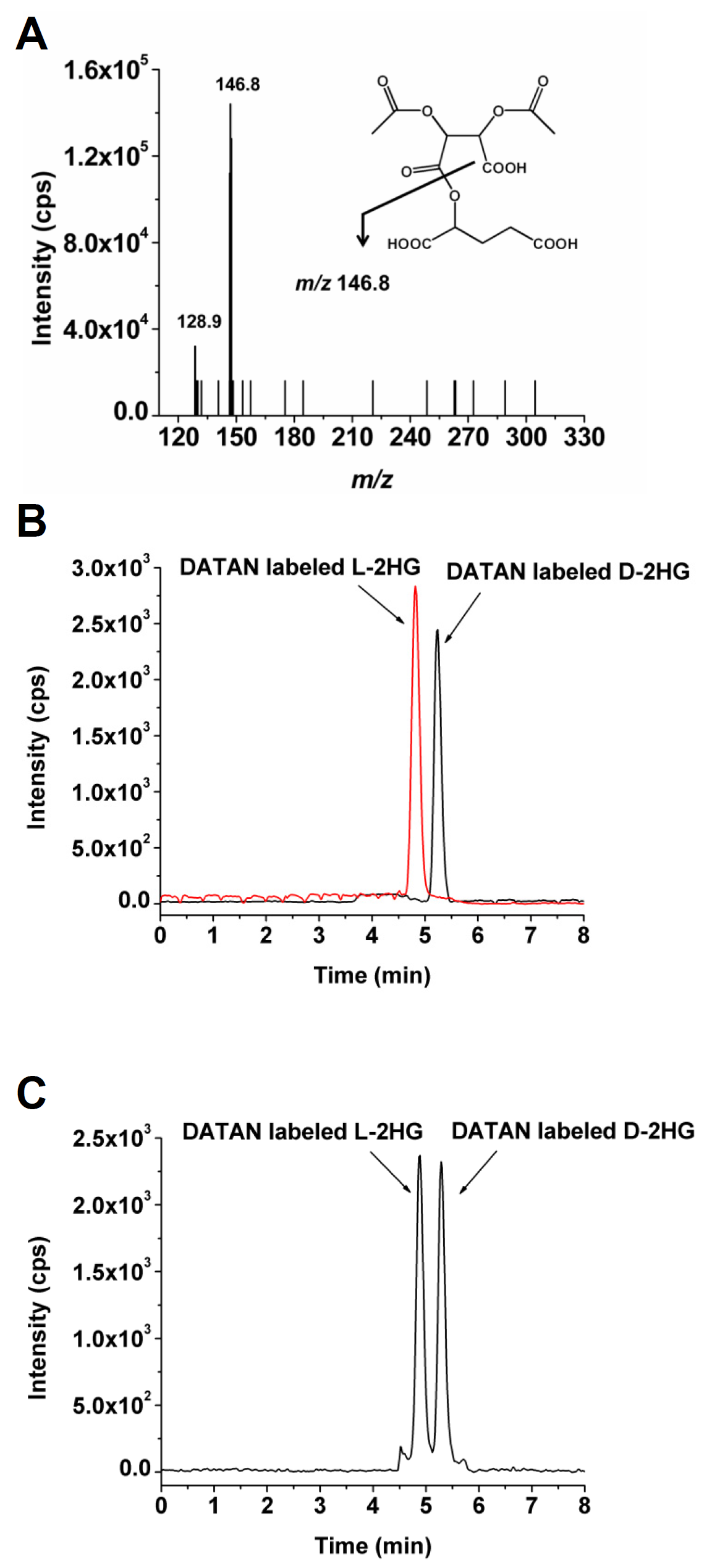


**Figure S2**. (A) The product ion spectrum of DATAN labeled 2-HG. (B) Extracted-ion chromatograms of DATAN labeled D-2HG and L-2HG standards. (C) Extracted-ion chromatogram of the mixture of DATAN labeled D-2HG and L-2HG standards.
